# Associations among oral health-related quality of life, subjective symptoms, clinical status, and self-rated oral health in Japanese university students: a cross-sectional study

**DOI:** 10.1186/s12903-016-0322-9

**Published:** 2016-11-30

**Authors:** Mayu Yamane-Takeuchi, Daisuke Ekuni, Shinsuke Mizutani, Kota Kataoka, Ayano Taniguchi-Tabata, Tetsuji Azuma, Michiko Furuta, Takaaki Tomofuji, Yoshiaki Iwasaki, Manabu Morita

**Affiliations:** 1 Departments of Preventive Dentistry, Okayama University Graduate School of Medicine, Dentistry and Pharmaceutical Sciences, 2-5-1 Shikata-cho, Kita-ku, Okayama 700-8558 Japan; 2 Section of Geriatric Dentistry Department of General Dentistry, Fukuoka Dental College, Tamura 2-15-1, Fukuoka, 814-0193 Japan; 3 Section of Preventive and Public Health Dentistry, Division of Oral Health, Growth and Development, Kyushu University Faculty of Dental Science, Fukuoka, 812-8582 Japan; 4 Health Service Center, Okayama University, 2-1-1 Tsushima-naka, Kita-ku, Okayama 700-8530 Japan

**Keywords:** Self-rated oral health, Oral health-related quality of life, Temporomandibular disorders, Stomatitis, DMFT, Malocclusion

## Abstract

**Background:**

The present study aimed to elucidate the associations among self-rated oral health, clinical oral health status, oral health behaviors, subjective oral symptoms, and oral health-related quality of life (OHRQoL) in a group of Japanese university students.

**Methods:**

Of 2051 participants, 2027 (98.83%) students received an optional oral examination and answered a questionnaire including items regarding age, sex, self-rated oral health, oral health behaviors, subjective oral symptoms, and OHRQoL [The Oral Health Impact Profile (OHIP)-14]. On oral examination, the decayed, missing, and filled teeth (DMFT) score, Community Periodontal Index (CPI), the percentage of teeth showing bleeding on probing (%BOP), and malocclusion were recorded. Structural equation modelling (SEM) analysis was used to test associations.

**Results:**

The mean score (± SD) of OHIP-14 was 1.92 ± 5.47. In the SEM analysis, the final model showed that self-rated oral health, oral pain, malocclusion, and the DMFT score were directly associated with the OHRQoL, and subjective symptoms of temporomandibular disorders (TMD) and recurrent aphthous stomatitis were both directly and indirectly associated (*p* < 0.05). CPI, %BOP, and oral health behaviors were excluded from the final model.

**Conclusions:**

OHRQoL was associated with self-related oral health, subjective symptoms of TMD, oral pain and stomatitis, DMFT, and malocclusion in this group of Japanese university students.

## Background

Oral health-related quality of life (OHRQoL) represents the subjective experience of symptoms related to oral conditions that have an impact on well-being [1, 2]. OHRQoL is an important part of the Global Oral Health Program [3]. Theoretically, OHRQoL is a function of various symptoms and experiences and represents the person’s subjective perspective. Dental clinical status has been shown to influence OHRQoL in older adults over a 3-month reference period [2]. For example, upper or lower denture need and decayed, missing, and filled teeth (DMFT) cause poor OHRQoL [2]. The plausibility for the association may be related to the impaired function caused by missing teeth. Periodontal disease also affects OHRQoL [4–6]. The relationship between dental clinical status and OHRQoL has also been demonstrated in some community-based studies including elderly and young populations [6–14]. However, few studies have investigated the relationship between dental clinical status and OHRQoL in the young population in Japan.

Young university students are in a dynamic transition period of growth and development that bridges adolescence (high school students) and adulthood (people in the community) [15, 16]. Since many of them are living away from home for the first time in their lives, their health, lifestyle, and behaviors could be easily changed [16]. When their oral health behaviors deteriorate, their clinical status can easily become exacerbated. Furthermore, poor oral health behaviors, such as high sugar consumption and inadequate tooth-brushing habits, may lead to adverse effects on OHRQoL [17, 18].

Self-rated oral health is often assessed in epidemiologic studies, since it permits easy evaluation of the participants’ general oral health condition. Based on the results of previous studies, information on self-rated oral health should be included during surveillance of oral health in young people [16, 19]. In addition, an association between self-rated oral health and OHRQoL has been identified in elderly people [10]. Although previous studies have investigated self-rated oral health and OHRQoL in adults [20–22], the association in the young population remains unknown.

We hypothesized that university students with better self-rated oral health, clinical status, and oral health behaviors, and less subjective oral symptoms would report better OHRQoL. Therefore, the aim of the present study was to elucidate the associations among OHRQoL, self-rated oral health, clinical status, oral health behaviors, and subjective oral symptoms in a group of Japanese university students.

## Methods

The first-year students of all departments of Okayama University underwent a mandatory general health examinations at the Health Service Center in April 2014. Of 2051 participants, 2027 (98.83%) students underwent an optional oral examination and completed a questionnaire. The exclusion criteria included incomplete data and age ≥20 years. To avoid the influence of age-related factors, most of the participants were 18 or 19 years old [16, 23]. The protocol of this study was approved by the Ethics Committee of Okayama University Graduate School of Medicine, Dentistry and Pharmaceutical Sciences (No.808). Verbal consent was obtained from all participants.

### Questionnaire

All participants were mailed a questionnaire prior to the health examination. The questionnaire included the following items: age, sex, general condition, self-rated oral health, oral health behaviors, subjective oral symptoms, and OHRQoL.

The question ‘In general, how do you consider your oral health?’ was used to assess self-rated oral health; the responses included ‘very good’, ‘good’, ‘fair’, ‘poor’, or ‘very poor’ [16].

Oral health behavior was assessed by asking about the daily frequency of tooth-brushing, with the possible responses being 1, 2, or ≥3 times. Participants were also asked to indicate whether they used dental floss and had a regular dental check-up during the past year [23].

With respect to subjective oral symptoms, the presence of oral pain and recurrent aphthous stomatitis during the past 3 months was answered as either yes or no [16]. Questions related to temporomandibular disorder (TMD) symptoms included: During the past year, 1) have you ever noticed any sound around your ears? (clicking), 2) Have you ever felt pain around the temporomandibular joint (TMJ) while opening your mouth or chewing food? (pain in TMJ), and 3) Have you ever had difficulty opening your mouth? (difficulty opening mouth) [3]. The response options for each question were frequently, sometimes, rarely, or never [16, 24].

The Japanese version of Oral Health Impact Profile (OHIP)-14 was selected to evaluate OHRQoL. The OHIP-14 is a short version of the OHIP-49 [25]. The OHIP-14 includes 14 items that explore the following seven dimensions: functional limitation, physical pain, psychological discomfort, physical disability, social disability, and handicap. Response options are “often = 4”, “fairly often = 3”, “occasionally = 2”, “hardly ever = 1”, or “never = 0” [26]. The higher the score on the OHIP-14, the higher the impact of oral conditions on OHRQoL.

### Oral examination

Five dentists (MY, DE, TA, SM, and KK) recorded the oral health status of the participants. The DMFT score was used to evaluate dental caries status based on the World Health Organization caries diagnostic criteria [27]. Periodontal condition was assessed using the Community Periodontal Index (CPI) [27]. Ten teeth were selected for periodontal examination: two molars in each posterior sextant and the upper right and lower left central incisors. Measurements were made using a CPI probe (YDM, Tokyo, Japan) at six sites (mesio-buccal, mid-buccal, disto-buccal, disto-lingual, mid-lingual, and mesio-lingual) per tooth. The percentage of teeth exhibiting bleeding on probing (%BOP) was also calculated in the same teeth examined for the CPI [16]. After training of the examiners, the DMFT score and probing pocket depth were recorded and repeated within a 2-week interval in three volunteers. Intra- and inter-examiner agreements for the oral examination were good, as indicated by kappa statistics of more than 0.8.

A modified version of the Index of Orthodontic Treatment Need (IOTN) was used to assess malocclusion [28]. Based on the results of a previous study, the modified IOTN appears to be a useful tool for non-specialists to screen for malocclusion in oral health surveys [29]. The dental health component of the modified IOTN is graded as either 0 or 1, with 0 = no definite need for orthodontic treatment and 1 = definite need for orthodontic treatment), with no subcategories. The modified IOTN was assessed by five dentists who were not orthodontists. A preliminary check showed that the kappa value was greater than 0.80.

### Statistical analyses

**Fig. 1:**
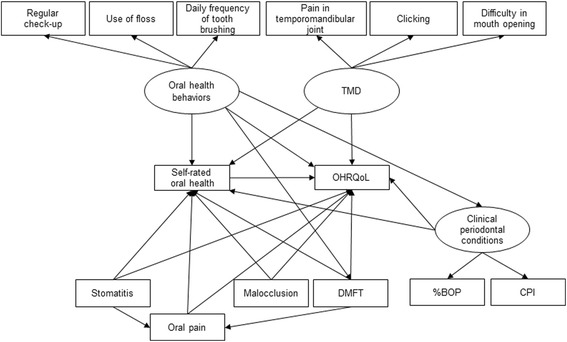
Ideal model. Ideal model showing the associations among self-rated oral health, subjective symptoms, clinical status, oral health behaviors, and OHRQoL. Rectangles indicate observed variables, and ovals show latent variables. The values of single-headed arrows indicate the standardized coefficients. BOP, bleeding on probing; CPI, Community Periodontal Index; DMFT, decayed, missing, and filled teeth; OHRQoL, oral health-related quality of life; TMD, temporomandibular disorders

Structural equation modelling (SEM) analysis was used to test the associations among self-rated oral health, clinical oral health status, oral health behaviors, subjective oral symptoms, and OHRQoL [16, 30, 31]. Based on our hypothesis, an ideal model about the association between self-rated oral health, OHRQoL, and the other variables is shown in Fig. 1. SEM enables variables to act both as independent and dependent variables and has some advantage over multiple-regression techniques for analyzing relationships within a conceptual model by allowing the inclusion of latent variables. Latent variables are those that cannot be measured directly but are estimated from measured variables in the model. The present data included continuous variables and several dichotomous variables and those with three or four categories. Therefore, the path analysis was performed using weighted least-squares parameter estimates (WLSMV). WLSMV uses a diagonal weight matrix with robust standard errors and mean- and variance-adjusted chi-square test statistics. The associations were assessed using M plus version 6 (Muthén & Muthén, Los Angeles, CA, USA.). For the global fit indices, a non-significant chi-square indicates that the data do not differ significantly from the hypotheses represented by the model; for the comparative fit index (CFI) and Tucker-Lewis index (TLI), fit indices of above 0.90 (preferably above 0.95) indicate a well-fitting model [32, 33]. For root mean square error of approximation (RMSEA), a fit of less than 0.05 indicates a well-fitting model [32, 34]. A significance level of *p* < 0.05 was used for the regression coefficients. “TMD”, “Oral health behaviors”, and “Clinical periodontal conditions” were set as latent variables. The latent construct “TMD” comprised “Pain in TMJ”, “Clicking,” and “Difficulty opening mouth”; “Oral health behaviors” included “Frequency of tooth brushing”, “Regular check-up”, and “Use of floss”; and “Clinical periodontal conditions” included the “CPI” and “%BOP”. Low scores indicated a good condition, that is, for self-rated oral health “1 = very good”, “2 = good”, “3 = fair”, “4 = poor”, and “5 = very poor”; for TMD, “1 = never”, “2 = rarely”, “3 = sometimes”, and “4 = frequently”; and for tooth brushing frequency, “1 = three times or more”, “2 = two times”, and “3 = 1 time or less”. For other parameters, “1 = yes” and “2 = no”.

Cohen’s effect size was assessed using correlation coefficients or the standardized coefficient that corresponded to r; small, medium, and large effect sizes were 0.10, 0.30, and 0.50, respectively [32].

## Results

A total of 243 participants who had provided incomplete data in their questionnaires and 126 participants who were 20 years or older were excluded. As a result, data from 1901 students (1095 males, 806 females) were analyzed. Table 1 shows the characteristics of the participants. Overall, 358 (18.8%) participants had poor self-rated oral health. On the OHIP-14, most participants reported a score of 0 as the total score (69.8%).

**Table 1:** Characteristics of participants (*n* = 1901)

Variable		
Male		1,095 (57.6)^a^
Self-rated oral health	Very good	218 (11.5)
	Good	481 (25.3)
	Fair	844 (44.4)
	Poor	312 (16.4)
	Very poor	46 (2.4)
Oral health behavior		
Regular check-up	Yes	318 (16.7)
Use of floss	Yes	243 (12.8)
Daily frequency of tooth-brushing	1 time	254 (13.4)
	2 times	1,402 (73.8)
	3 times or more	245 (12.9)
Subjective oral symptom		
Oral pain	Yes	53 (2.8)
Recurrent aphthous stomatitis	Yes	404 (21.3)
Temporomandibular disorders		
Pain in temporomandibular joint	Never	1,490 (78.4)
	Rarely	251 (13.2)
	Sometimes	132 (6.9)
	Frequency	28 (1.5)
Clicking	Never	1,072 (56.4)
	Rarely	348 (18.3)
	Sometimes	256 (13.5)
	Frequency	225 (11.8)
Difficulty in mouth opening	Never	1,521 (80.0)
	Rarely	190 (10.0)
	Sometimes	132 (6.9)
	Frequency	58 (3.1)
Clinical status		
Percentage of bleeding on probing		33.65±27.96^b^
Community Periodontal Index	0	249 (13.1)
	1	431 (22.7)
	2	928 (48.8)
	3	289 (15.2)
	4	4 (0.2)
Malocclusion	+	521 (27.4)
Decayed, missing, and filled teeth score		2.01±2.88
Oral Health Impact Profile-14		
Total		1.92±5.47
Functional limitation		0.23±0.84
Physical pain		0.36±1.09
Psychological discomfort		0.40±1.15
Physical disability		0.19±0.82
Psychological disability		0.33±1.03
Social disability		0.22±0.84
Handicap		0.20±0.81

^a^number (%)

^b^mean ± SD

**Fig. 2:**
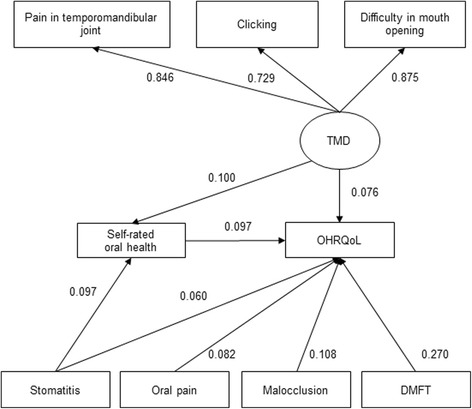
The final structural model. Rectangles indicate observed variables, and ovals show latent variables. The values of single-headed arrows indicate the standardized coefficients. All pathways are significant (*p* < 0.05). OHRQoL was associated with self-related oral health, subjective symptoms of TMD, oral pain and stomatitis, DMFT, and malocclusion. DMFT, decayed, missing, and filled teeth; OHRQoL, oral health-related quality of life; TMD, temporomandibular disorders

Figure 2 shows the parameters estimated for the final structural model. The value of chi-square was not significant (*χ* ^2^ = 25.582, df = 17, *P* = 0.1423). The CFI, TLI, and RMSEA values indicated good model-data fit (0.998, 0.996, and 0.013, respectively). The model showed that (1) self-rated oral health directly affected OHRQoL and the effect size was small, (2) the DMFT score directly affected OHRQoL and the effect size was medium, (3) subjective symptoms of TMD, oral pain and stomatitis, and malocclusion directly affected OHRQoL and the effect size was small. All pathways were significant (*p* < 0.05). Clinical periodontal conditions and oral health behaviors were excluded from the final model.

## Discussion

In the SEM analysis, self-related oral health, subjective symptoms (stomatitis, oral pain and TMD), and clinical status (malocclusion and DMFT) were positively associated with OHRQoL in university students. OHRQoL represents the subjective experience of symptoms related to oral conditions that have an impact on well-being [1, 2]. Many researchers have focused on the association between OHRQoL and other factors in the elderly population [2, 7–10]. To the best of our knowledge, this is the first study that showed associations among self-related oral health, subjective symptoms, clinical status, and OHRQoL among Japanese university students. In Japan, school health authorities have recently shown an interest in improvement of QoL for health promotion. To ensure the health of students, it is important to determine the predictors of OHRQoL, as in the present study that showed that self-related oral health, subjective symptoms (stomatitis, oral pain and TMD), and clinical status (malocclusion and DMFT) could be predictors of OHRQoL. Not only early detection of dental diseases, but also control of self-related oral health would contribute to improving OHRQoL and well-being among young people.

As for the characteristics of participants, the mean score (± SD) of OHIP-14 was 1.92 ± 5.47 in this study. It was relatively low compared to those in elderly people [8–11, 35]. In fact, 1326 participants (69.8%) had a score of 0. On the other hand, 358 (18.8%) participants had poor self-rated oral health that the value was lower than the data of Japanese university students (21.5–27.1%) in previous studies [16, 30]. The mean DMFT score (±SD) was 2.01 ± 2.88 in the participants aged 18 and 19 years. The mean DMFT score in this study was within the same range in previous studies comprising young adults aged 15 to 24 years in France [36] or four developed countries [37]. However, it was lower than that reported in a Japanese national survey in 2005 (3.2 ± 3.9 for those aged 15–19 years) [38].

Self-rated oral health was directly associated with OHRQoL, that is, poor self-related oral health resulted in poor OHRQoL, although the effect size of the path was small. The association was also observed in previous studies [10, 20–22], which support the present results. Self-rated oral health is assessed frequently in epidemiological studies for evaluation of the general oral health condition. Since assessment of self-rated oral health is relatively simple, which makes it easy to collect dental information in surveys [39], self-rated oral health as a predictor of OHRQoL in the young population may be useful in epidemiological studies.

With respect to clinical status, the DMFT score and malocclusion were directly associated with OHRQoL, that is, participants with a high DMFT score and malocclusion thought that they actually had poor OHRQoL. These results were similar to those of other studies [40–43]. Furthermore, the effect size of the path from the DMFT was medium and the highest of all parameters. Therefore, a decrease in the DMFT score can be most effective in changing OHRQoL. This concept was supported by a previous study suggesting that DMFT was a major predictor of low oral health-related quality of life in children [40]. The mechanisms are not clear, but there may be potential interactions. A high DMFT score is correlated with anxiety [44]. Dental treatment contributes by improving oral health status, anxiety, and OHRQoL [45] Thus, anxiety associated with caries experience might strongly influence OHRQoL.

Subjective symptoms (TMD, stomatitis, and oral pain) were associated with OHRQoL in university students. These findings were supported by previous studies in other populations [46–50]. Symptoms of TMD and stomatitis were also related to self-rated oral health. We previously reported a similar association [16]. Taken together, since the two symptoms directly and indirectly contribute to poor OHRQoL, control of TMD and stomatitis may be important for improving OHRQoL in the young population.

The path form oral health behaviors to OHRQoL did not fit the final model. However, the previous studies suggest a possible association between oral health behaviors and OHRQoL [51–53]. The discrepancy between this study and the previous studies may be explained by the difference in study populations and age (Japanese university students aged 18 and 19 years vs. Korean elderly aged 65–85 years, Brazilian orthodontic patients aged 14 to 30 years, or Spanish dental patients aged 18 to 87 years).

Clinical periodontal conditions such as the CPI score and %BOP were excluded from the final model and did not show a significant effect on OHRQoL in this study. The reason may be that young students with periodontal diseases generally have minimal symptoms. However, in periodontitis patients who have evident periodontal symptoms, OHRQoL was associated with clinical status [4–6]. Thus, prevention of periodontal disease in younger populations may require earlier detection of the disease or advice from specialists during routine oral examination [54].

In the present study, the OHIP-14 scores were relatively low and all effect sizes of the paths, except for DMFT to OHRQoL, may be low. However, exploring the association between OHRQoL and other factors should not be neglected, even though the score was not relatively high. Early detection and early treatment of oral diseases are important for improving OHRQoL in young people, as well as in elderly people. Since oral examinations are not mandatory for university students in Japan, routine health examinations in universities should include an investigation of predictors of OHRQoL.

The OHIP-14 was selected as an indicator of OHRQoL because the OHIP-14 is better at detecting psychosocial impacts among individuals and groups and more closely matches the main criteria for measurement of OHRQoL [55, 56]. Furthermore, the OHIP-14 is a shorter version of the OHIP-49 described by Slade and Spencer [57], but it retains the original conceptual dimensions contained in the OHIP-49. Thus, the OHIP-14 has the advantage of convenience compared to the full version for investigating OHRQoL in epidemiological studies.

In the SEM analysis, individual characteristics (sex) and environmental characteristics (socioeconomic factors) were not included. These factors were associated with QOL in Wilson and Cleary’s conceptual model [58]. When the data of males and females were separated in the SEM analysis, the findings in males and females were similar (data not shown). Thus, sex may not have affected the present results. On the other hand, it was not possible to investigate socioeconomic status in this study, which was a limitation. However, the socioeconomic status of students in this study may not vary as much as is observed in other countries, such as Brazil [59] and Tanzania [60], because only national university students were recruited.

This study has other limitations. First, it was a cross-sectional study, and to develop a better model, a prospective cohort study and an interventional study would be required. Second, the participants were all college freshmen at Okayama University, which may limit the generalizability of the findings to young Japanese students.

## Conclusions

The OHIP-14 score was positively associated with self-related oral health, subjective symptoms of TMD, oral pain and stomatitis, and clinical status (DMFT and malocclusion) in this group of Japanese university students.

## Abbreviations

%BOP: The percentage of teeth exhibiting bleeding on probing
CFI: Comparative fit index
CPI: Community Periodontal Index
DMFT: Decayed, missing, and filled teeth
IOTN: Index of Orthodontic Treatment Need
OHIP: The Oral Health Impact Profile
OHRQoL: Oral health-related quality of life
RMSEM: Root mean square error of approximation
SEM: Structural equation modelling
TLI: Tucker-Lewis index
TMD: Temporomandibular disorders
TMJ: Temporomandibular joint
WLSMV: Weighted least-squares parameter estimates

## References

[1] Locker D, Allen FP (2007). What do measures of ‘oral health-related quality of life’ measure?. Community Dent Oral Epidemiol.

[2] Rebelo MA, Cardoso EM, Robinson PG, Vettore MV (2016). Demographics, social position, dental status and oral health-related quality of life in community-dwelling older adults. Qual Life Res.

[3] Sischo L, Broder HL (2011). Oral health-related quality of life: what, why, how, and future implications. J Dent Res.

[4] Al-Harthi LS, Cullinan MP, Leichter JW, Thomson WM (2013). The impact of periodontitis on oral health-related quality of life: a review of the evidence from observational studies. Aust Dent J.

[5] Eltas A, Uslu MO, Eltas SD (2016). Association of oral health-related quality of life with periodontal status and treatment needs. Oral Health Prevent Dent.

[6] Buset SL, Walter C, Friedmann A, Weiger R, Borgnakke WS, Zitzmann NU (2016). Are periodontal diseases really silent? A systematic review of their effect on quality of life. J Clin Periodontol.

[7] Akifusa S, Soh I, Ansai T, Hamasaki T, Takata Y, Yohida A, Fukuhara M, Sonoki K, Takehara T (2005). Relationship of number of remaining teeth to health-related quality of life in community dwelling elderly. Gerodontology.

[8] Slade GD, Spencer AJ (1994). Social impact of oral conditions among older adults. Aust Dent J.

[9] Slade GD, Spencer AJ, Locker D, Hunt RJ, Strauss RP, Beck JD (1996). Variations in the social impact of oral conditions among older adults in South Australia, Ontario, and North Carolina. J Dent Res.

[10] Mariño R, Schofield M, Wright C, Calache H, Minichiello V (2008). Self-reported and clinically determined oral health status predictors for quality of life in dentate older migrant adults. Community Dent Oral Epidemiol.

[11] Einarson S, Gerdin EW, Hugoson A (2014). Oral health-related quality of life and its relationship to self-reported oral discomfort and clinical status. Swed Dent J.

[12] Daly B, Newton JT, Fares J, Chiu K, Ahmad N, Shirodaria S, Bartlett D (2011). Dental tooth surface loss and quality of life in university students. Prim Dent Care.

[13] Yiengprugsawan V, Somkotra T, Seubsman SA, Sleigh AC, Thai Cohort Study Team (2011). Oral Health-Related Quality of Life among a large national cohort of 87,134 Thai adults. Health Qual Life Outcomes.

[14] Batista MJ, Perianes LB, Hilgert JB, Hugo FN, Sousa ML (2014). The impacts of oral health on quality of life in working adults. Braz Oral Res..

[15] Wei CN, Harada K, Ueda K, Fukumoto K, Minamoto K, Ueda A (2012). Assessment of health-promoting lifestyle profile in Japanese university students. Environ Health Prev Med.

[16] Kojima A, Ekuni D, Mizutani S, Furuta M, Irie K, Azuma T, Tomofuji T, Iwasaki Y, Morita M (2013). Relationships between self-rated oral health, subjective symptoms, oral health behavior and clinical conditions in Japanese university students: a cross-sectional survey at Okayama University. BMC Oral Health.

[17] Krisdapong S, Prasertsom P, Rattanarangsima K, Sheiham A (2013). Sociodemographic differences in oral health-related quality of life related to dental caries in thai school children. Community Dent Health.

[18] Broadbent JM, Zeng J, Foster Page LA, Baker SR, Ramrakha S, Thomson WM (2016). Oral health-related beliefs, behaviors, and outcomes through the life course. J Dent Res.

[19] Ostberg AL, Eriksson B, Lindblad U, Halling A (2003). Epidemiological dental indices and self-perceived oral health in adolescents: ecological aspects. Acta Odontol Scand.

[20] Luo Y, McGrath C (2008). Oral health and its impact on the life quality of homeless people in Hong Kong. Community Dent Health.

[21] Dahl KE, Wang NJ, Skau I, Ohrn K (2011). Oral health-related quality of life and associated factors in Norwegian adults. Acta Odontol Scand.

[22] Gonzales-Sullcahuamán JA, Ferreira FM, de Menezes JV, Paiva SM, Fraiz FC (2013). Oral health-related quality of life among Brazilian dental students. Acta Odontol Latinoam.

[23] Furuta M, Ekuni D, Irie K, Azuma T, Tomofuji T, Ogura T, Morita M (2011). Sex differences in gingivitis relate to interaction of oral health behaviors in young people. J Periodontol.

[24] Akhter R, Morita M, Esaki M, Nakamura K, Kanehira T (2011). Development of temporomandibular disorder symptoms: a 3-year cohort study of university students. J Oral Rehabil.

[25] Slade GD, Spencer AJ (1994). Development and evaluation of the Oral Health Impact Profile. Community Dent Health.

[26] Ide R, Yamamoto R, Mizoue T (2006). The Japanese version of the Oral Health Impact Profile (OHIP)--validation among young and middle-aged adults. Community Dent Health.

[27] World Health Organization: Oral Health survey, Basic methods. 4th edition. http://apps.who.int/iris/bitstream/10665/41905/1/9241544937.pdf. 1997. Accessed 25 May 2016.

[28] Kataoka K, Ekuni D, Mizutani S, Tomofuji T, Azuma T, Yamane M, Kawabatat Y, Iwasaki Y, Morita M (2015). Association between self-reported bruxism and malocclusion in university students: a cross-sectional study. J Epidemiol.

[29] Ekuni D, Furuta M, Irie K, Azuma T, Tomofuji T, Murakami T, Yamashiro T, Ogura T, Morita M (2011). Relationship between impacts attributed to malocclusion and psychological stress in young Japanese adults. Eur J Orthod.

[30] Furuta M, Ekuni D, Takao S, Suzuki E, Morita M, Kawachi I (2012). Social capital and self-rated oral health among young people. Community Dent Oral Epidemiol.

[31] Kile ML, Hoffman E, Rodrigues EG, Breton CV, Quamruzzaman Q, Rahman M, Mahiuddin G, Hsueg YM, Christiano DC (2011). A pathway-based analysis of urinary arsenic metabolites and skin lesions. Am J Epidemiol.

[32] Mizutani S, Ekuni D, Tomofuji T, Irie K, Azuma T, Iwasaki Y, Morita M (2015). Self-efficacy and progression of periodontal disease: a prospective cohort study. J Clin Periodontol.

[33] Hu L-t, Bentler PM (1999). Cutoff criteria for fit indices in covariance structure analyses: conventional criteria versus new alternatives. Struct Equ Model.

[34] Browne MW, Cudeck R, Bollen KA, Long JS (1993). Alternative ways of assessing model fit. Testing structural equation models.

[35] Locker D, Clarke M, Payne B (2000). Self-perceived oral health status psychological well-being, and life satisfaction in an older adult population. J Dent Res.

[36] Bou C, Miquel JL, Poisson P (2006). Oral health status of 1500 university students in Toulouse France. Odontostomatol Trop.

[37] Bernabé E, Sheiham A (2014). Age, period and cohort trends in caries of permanent teeth in four developed countries. Am J Public Health.

[38] Kawashita Y, Kitamura M, Saito T (2012). Monitoring time-related trends in dental caries in permanent teeth in Japanese national surveys. Int Dent J.

[39] Astrom AN, Mashoto K (2002). Determinants of self-rated oral health status among school children in northern Tanzania. Int J Paediatr Dent.

[40] Alsumait A, ElSalhy M, Raine K, Cor K, Gokiert R, Al-Mutawa S, Amin M (2015). Impact of dental health on children’s oral health-related quality of life; a cross-sectional study. Health Qual Life Outcomes.

[41] Bastos RS, Carvalho ES, Xavier A, Caldana ML, Bastos JR, Lauris JR (2012). Dental caries related to quality of life in two Brazilian adolescent groups: a cross-sectional randomised study. Int Dent J.

[42] Palomares NB, Celeste RK, Miguel JA (2016). Impact of orthosurgical treatment phases on oral health-related quality of life. Am J Orthod Dentofacial Orthop.

[43] Choi SH, Kim JS, Cha JY, Hwang CJ (2016). Effect of malocclusion severity on oral health-related quality of life and food intake ability in a Korean population. Am J Orthod Dentofacial Orthop.

[44] Samorodnitzky GR, Levin L (2005). Self-assessed dental status, oral behavior, DMF, and dental anxiety. J Dent Educ.

[45] Vermaire JH, de Jongh A, Aartman IH (2008). Dental anxiety and quality of life: the effect of dental treatment. Community Dent Oral Epidemiol.

[46] Almoznino G, Zini A, Zakuto A, Sharav Y, Haviv Y, Hadad A, Chweidon H, Yarom N, Benoliel R (2015). Oral health-related quality of life in patients with temporomandibular disorders. J Oral Facial Pain Headache.

[47] Blanco-Aguilera A, Blanco-Hungría A, Biedma-Velázquez L, Serrano-del-Rosal R, González-López L, Blanco-Aguilera E, Segura-Saint-Gerons R (2014). Application of an oral health-related quality of life questionnaire in primary care patients with orofacial pain and temporomandibular disorders. Med Oral Patol Oral Cir Bucal.

[48] McGrath C, Comfort MB, Lo EC, Luo Y (2003). Patient-centred outcome measures in oral surgery: validity and sensitivity. Br J Oral Maxillofacial Surg.

[49] Hapa A, Aksoy B, Polat M, Aslan U, Atakan N (2011). Does recurrent aphthous stomatitis affect quality of life? A prospective study with 128 patients evaluating different treatment modalities. J Dermatolog Treat.

[50] Dahlström L, Carlsson GE (2010). Temporomandibular disorders and oral health-related quality of life. A systematic review. Acta Odontol Scand.

[51] Yoon HS, Kim HY, Patton LL, Chun JH, Bae KH, Lee MO (2013). Happiness, subjective and objective oral health status, and oral health behaviors among Korean elders. Community Dent Oral Epidemiol.

[52] Zanatta FB, Author TM, Antoniazzi RP, Pinto TM, Rösing CK (2012). Association between gingival bleeding and gingival enlargement and oral health-related quality of life (OHRQoL) of subjects under fixed orthodontic treatment: a cross-sectional study. BMC Oral Health.

[53] Montero J, Albaladejo A, Zalba JI (2014). Influence of the usual motivation for dental attendance on dental status and oral health-related quality of life. Med Oral Patol Oral Cir Bucal.

[54] Ueno M, Zaitsu T, Ohara S, Wright C, Kawaguchi Y (2015). Factors influencing perceived oral health of Japanese middle- aged adults. Asia Pac J Public Health.

[55] Atchison KA, Dolan TA (1990). Development of the geriatric oral health assessment index. J Dent Educ.

[56] Locker D, Matear D, Stephens M, Lawrence H, Payne B (2001). Comparison of the GOHAI and OHIP-14 as measures of the oral health-related quality of life of the elderly. Community Dent Oral Epidemiol.

[57] Slade GD (1997). Derivation and validation of a short-form oral health impact profile. Community Dent Oral Epidemiol.

[58] Wilson IB, Cleary PD (1995). Linking clinical variables with health-related quality of life. A conceptual model of patient outcomes. JAMA.

[59] Paula JS, Leite IC, Almeida AB, Ambrosano GM, Pereira AC, Mialhe FL (2012). The influence of oral health conditions, socioeconomic status and home environment factors on schoolchildren’s self-perception of quality of life. Health Qual Life Outcomes.

[60] Mbawalla HS, Masalu JR, Astrøm AN (2010). Socio-demographic and behavioural correlates of oral hygiene status and oral health related quality of life, the Limpopo-Arusha school health project (LASH): a cross-sectional study. BMC Pediatr.

